# Mechanisms of the Procognitive Effects of Xanthotoxin and Umbelliferone on LPS-Induced Amnesia in Mice

**DOI:** 10.3390/ijms22041779

**Published:** 2021-02-10

**Authors:** Łukasz Kurach, Sylwia Kulczycka-Mamona, Joanna Kowalczyk, Krystyna Skalicka-Woźniak, Anna Boguszewska-Czubara, Nesrine El Sayed, Mitat Osmani, Karol Iwaniak, Barbara Budzyńska

**Affiliations:** 1 Independent Laboratory of Behavioral Studies, Medical University of Lublin, 4A Chodzki Str., 20-093 Lublin, Poland; barbara.budzynska@umlub.pl; 2 Department of Applied Pharmacy, Medical University of Lublin, 1 Chodzki Str., 20-093 Lublin, Poland; sylwia.kulczycka-mamona@umlub.pl (S.K.-M.); joanna.kowalczyk19@gmail.com (J.K.); karol.iwaniak@umlub.pl (K.I.); 3 Independent Laboratory of Natural Products Chemistry, Medical University of Lublin, 1 Chodzki Str., 20-093 Lublin, Poland; kskalicka@pharmacognosy.org; 4 Department of Medicinal Chemistry, Medical University of Lublin, 4A Chodzki Str., 20-093 Lublin, Poland; anna.boguszewska-czubara@am.lublin.pl; 5 Department of Pharmacology and Toxicology, Faculty of Pharmacy, Cairo University, Cairo 11562, Egypt; nesrine.salah@pharma.cu.edu.eg; 6 Department of Pharmacy, University of Pristina, St. Bulevardi i Dëshmorëve, 10000 Pristina, Kosovo; mitatosmani3@gmail.com

**Keywords:** coumarins, memory impairment, neuroinflammation

## Abstract

Neuroinflammation plays an essential role in the pathogenesis of neurodegenerative diseases such as Alzheimer’s disease. Although coumarins have been shown to improve cognitive function in animal models and exert anti-inflammatory effects in cell cultures, the exact mechanism of their neuroprotective effects has not yet been fully elucidated. The present study aimed to investigate the neuroprotective effects of xanthotoxin (furanocoumarin) and umbelliferone (simple coumarin) in lipopolysaccharide-induced cognitive dysfunction in mice. For evaluation memory and learning processes, a passive avoidance test was used. Furthermore, acetylcholinesterase level and impact on the tumor necrosis factor α, interleukin 10 levels in the whole brain, and cyclooxygenase-II in hippocampus was established. Subchronic administration of both coumarins (15 mg/kg) enhanced the learning and memory function, but only the xanthotoxin improved cognitive processes impaired by lipopolysaccharide (0.8 mg/kg) administration. Behavioral results stay in line with acetylcholinesterase level in the brain. A statistically significant decrease in the level of tumor necrosis factor α and cyclooxygenase-II in lipopolysaccharide-treated rodents after coumarins’ administration was observed. Together, our findings demonstrate that both coumarins improved cognitive functions, but only xanthotoxin significantly enhanced the learning and memory function and reduced the level of acetylcholinesterase in lipopolysaccharide-treated mice. This effect may suggest that only furanocoumarin—xanthotoxin attenuates neuroinflammation and enhances cholinergic neurotransmission, thus it can be a potential remedy with procognitive potential effective in treatment of neuroinflammatory disease.

## 1. Introduction

Dementia is a broad term for several diseases that affects either memory or cognitive abilities and complicate daily activities. According to the World Health Organization (WHO) report, this problem concerns nearly 50 million of world population, and around 10 million cases are diagnosed annually. Incidents are forecasted to increase to 75 million in 2030 and 132 million by 2050 [1]. One of the major problems may be that dementia is already well advanced at the time of diagnosis. There are risk factors like aging, family history, diabetes, hypertension, lifestyle, but there is still a lack of understanding of the etiology of dementia. Current treatment is based on reducing memory deterioration using cholinesterase inhibitors with NMDA receptor blockers [2,3]. Chronic and sustained neuroinflammation is a key factor in development and progression of neurodegenerative diseases such as Alzheimer’s disease (AD), the most common cause of dementia (60–70% of cases) [4,5]. Recent studies suggest an involvement of TNF-α in the pathophysiology of AD [6]. The elevated level of this cytokine was found in the brain tissue of either AD transgenic mice or patients with AD [7]. Also, deletion of tumor necrosis factor receptor (TNFR) type 1, but not TNFR type 2, inhibit cognitive deficits, Aβ formation and β-secretase 1 (BACE1) expression [8,9,10,11]. Clinical trials suggest that TNF-α inhibitors might slow down cognitive decline and improve daily activities in AD patients [12,13]. Therefore there is a need to look for new substances that could counteract the development of AD, penetrate the blood-brain barrier (BBB) efficiently during systematic administration, improve cognitive functions, and at the same time, have fewer side effects. 

Different animal models are used for evaluation of mechanisms and treatment of neurodegenerative diseases. Lipopolysaccharide (LPS)–induced neurodegeneration seems to mimic complex mechanisms based on neuroinflammation. It involves many pathways and signaling proteins, especially cytokines like tumor necrosis factor-α (TNF-α), interleukin-10 (IL-10) and cyclooxygenase-2 (COX-2), which are mediators controlling the response to inflammation [14]. Chronic neuroinflammation causes the degeneration of neurons, glia, and BBB, necessary for normal functioning. Too strong and non-controlled response from the immune system can compromise BBB, induce long term neuronal toxicity, memory impairment, and hence reduce neurogenesis [15]. 

**Figure 1:**
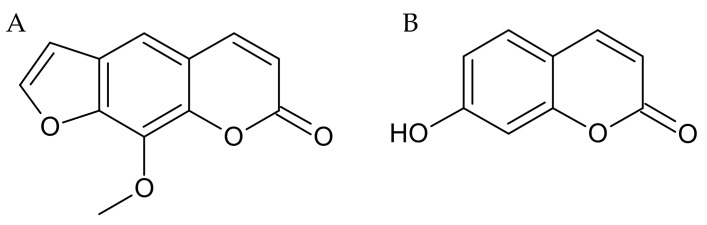
Chemical structure of (**A**) xanthotoxin and (**B**) umbelliferone.

Recently, considerable attention is focused on investigating the effectiveness of herbal products in the management of many neurodegenerative disorders. One of the promising substances that act towards the central nervous system (CNS) are coumarins, a large group of phytochemicals with a wide range of biological activities [16]. Xanthotoxin (a furanocoumarin) and umbelliferone (a simple coumarin) (Figure 1) are part of this family and can be found in *Apiaceae* and *Rutaceae* families [17]. 

Previously, xanthotoxin and umbelliferone have shown an inhibitory effect towards butyrylcholinesterase (BChE) and acetylcholinesterase (AChE) [18,19]. Moreover, xanthotoxin exhibits moderate antioxidant properties in FRAP assay [18] that may be important for neuroprotective effect against reactive oxygen species (ROS), resulting from prooxidant-antioxidant imbalance during chronic neuroinflammation. Our previous studies revealed that umbelliferone, xanthotoxin, and bergapten prolonged the anti-depressant and procognitive activity of nicotine [20]. Furthermore, imperatorin, C-8 substituted structural analog of xanthotoxin, exerted the anxiolytic and procognitive effects [21]. Improvement of memory and learning processes was revealed in scopolamine-induced dementia in the passive avoidance (PA) paradigm [22]. Similarly, xanthotoxin showed procognitive effects after acute and subchronic injections in scopolamine-induced memory disturbance in mice [23]. Based on our studies, we suggest that antioxidant properties, and cholinergic mechanisms are involved in these effects. However, memory loss is a multifactorial process. Therefore, searching for new compounds that improve memory processes and deepening knowledge about its mechanisms of action is crucial.

Thus, in the current study, we attempted to evaluate the effects of xanthotoxin (furanocoumarin) and umbelliferone (simple coumarin) treatment on LPS-induced neuroinflammation in Swiss male mice. For the evaluation of memory and learning processes, the PA test was used. Furthermore, we established cholinesterase level and the influence on the TNF-α, IL-10, and COX-2 levels in the brain. These mediators are involved in the control of inflammatory reaction, activate many pathways and therefore are good markers of it.

## 2. Results

### 2.1. Repeated Xanthotoxin and Umbelliferone Injections Effect on Memory-Related Processes Induced by LPS in the PA Test in Mice

**Figure 2:**
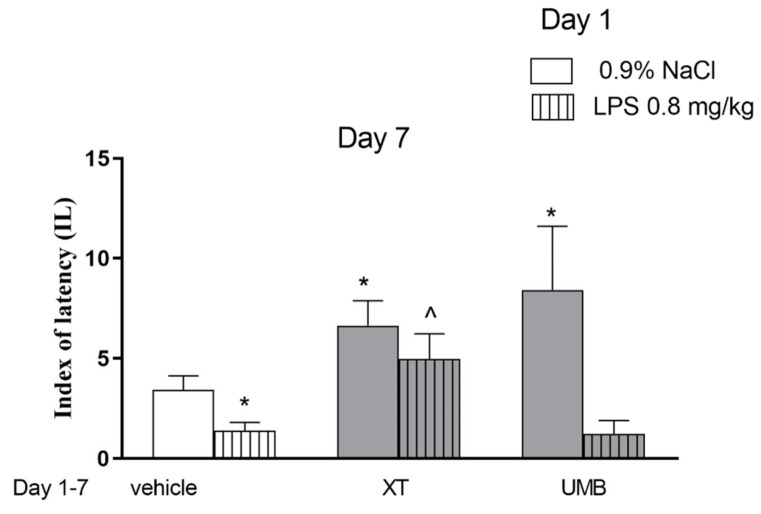
Effects of subchronic administration of xanthotoxin (XT, 15 mg/kg) and umbelliferone (UMB, 15 mg/kg, i.p.) on LPS (0.8 mg/kg)-induced impairment of memory acquisition trial using the PA test in mice. Mice were injected with XT and UMB 60 min after LPS administration and then consecutively for 6 days. On the seventh day coumarins were injected 30 min before the first trial and animals were retested 24 h after the last injection; *n* = 10; the means ± SEM; * *p* < 0.05; vs. saline-treated control group, ^^^ *p* < 0.05 vs. LPS-treated control group; Bonferroni’s test.

In order to check the influence of subchronic xanthotoxin and umbelliferone administration on the acquisition of the memory processes, the dose of 15 mg/kg as the active one in the PA test were selected. Figure 2 indicates the effects of repeated injections of both coumarins on memory acquisition impaired by LPS during the retention trial in the PA task (two-way ANOVA: pre-treatment [F(1.60) = 14.32; *p* = 0.0003], treatment [F(2.60) = 7.72; *p* = 0.009] and interactions effect [F(2.60) = 1.25; *p* = 0.2937]). The post hoc Bonferroni’s test revealed that umbelliferone (*p* < 0.05) and xanthotoxin (*p* < 0.05) given repeatedly, significantly increased IL value, as compared with the saline-treated mice, thus indicating that subchronic administration of tested compounds improved acquisition of the memory and learning processes during the retention trial. In contrast, the post hoc Bonferroni’s test revealed a statistically significant improvement in memory and learning processes in the animals administered with LPS (1st day, 0.8 mg/kg), and during next 6 days with xanthotoxin (15 mg/kg) (*p* < 0.05) vs. the LPS-treated mice, administered with saline during the next 6 days. Such activity was not observed for umbelliferone.

### 2.2. The Influence of Subchronic Administration of Xanthotoxin and Umbelliferone on Locomotor Activity

**Figure 3:**
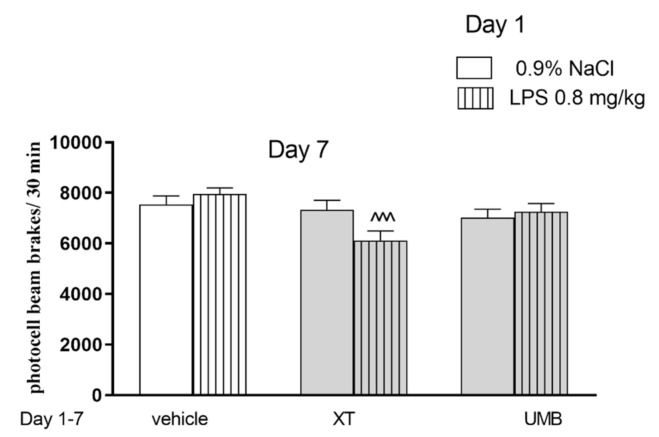
Effect of LPS (0.8 mg/kg, i.p.) and xanthotoxin (XT, 15 mg/kg) and umbelliferone (UMB, 15 mg/kg, i.p.) administered separately or in combination on spontaneous locomotor activity in mice. Mice were injected with XT and UMB 60 min after LPS administration and then consecutively for 6 days. On the seventh day immediately after coumarins injections mice were placed in actimeters. Locomotor activity (number of interruptions of light beams) was recorded for the 30 min. Data is presented as the means ± SEM. *n* = 9–10 ^^^ *p* < 0.001, vs. saline-treated group; (post hoc Bonferroni’s test).

Figure 3 indicates the effects of acute administration of LPS and repeated injections of xanthotoxin and umbelliferone on locomotor activity (two-way ANOVA: pre-treatment [F(1,59) = 1.61 *p* = 0.1192], treatment [F(2,59) = 8.33; *p* = 0.006] and interactions effect [F(2,59) = 3.71; *p* = 0.0301]). The post-hoc Bonferroni’s test confirmed that xanthotoxin at the dose of 15 mg/kg significantly decreased locomotor activity in mice injected with LPS in comparison with the LPS-treated mice (*p* < 0.001). No activity was observed for umbelliferone.

### 2.3. Subchronic Injection of Xanthotoxin and Umbelliferone on AChE Level Altered by LPS Administration

**Figure 4:**
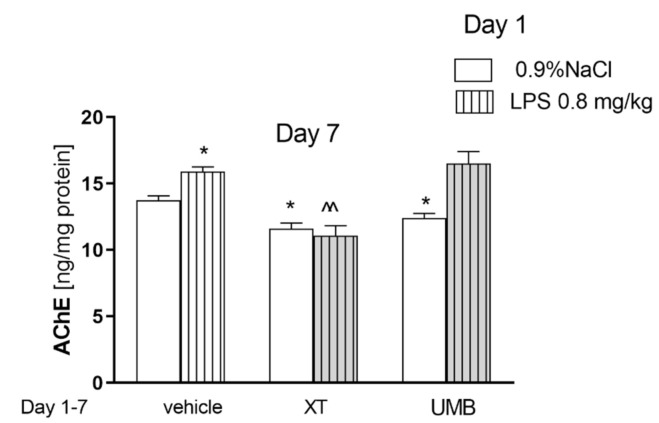
Effects of acute injection of LPS (0.8 mg/kg) and repeated xanthotoxin and umbelliferone (15 mg/kg) alone or in combination on AChE level in the brain. LPS (0.8 mg/kg, i.p.) was administered on the day 1st, XT and UMB were administered repeatedly (day 1–7). The brains were collected 24 h after the last injection; *n* = 8–10; the means ± SEM; * *p* < 0.05 vs. saline-treated control group, ^^ *p* < 0.01 vs. LPS-treated control group, Bonferroni’s test.

Figure 4 indicates the effects of acute injection of LPS (0.8 mg/kg) and repeated administration of xanthotoxin and umbelliferone (15 mg/kg) alone or in combination on AChE level measured in the brain (pretreatment [F(1.58) = 61.26, *p* < 0.0001], without interactions [F(2.58) = 1.67, *p* = 0.2095]) and treatment [F(2.58) = 0.03, *p* = 0.8598]; two-way ANOVA). A statistically significant increase in the AChE concentration in the brain was observed after a single injection of LPS (*p* < 0.05). Subchronic administration of both coumarins alone decreased the level of the enzyme (*p* < 0.05). A significant decrease in AChE concentration was also noticed in the brain after repeated administration of xanthotoxin (15 mg/kg) in mice with altered level of this enzyme observed after LPS administration (*p* < 0.01) vs. LPS-treated mice.

**Figure 5:**
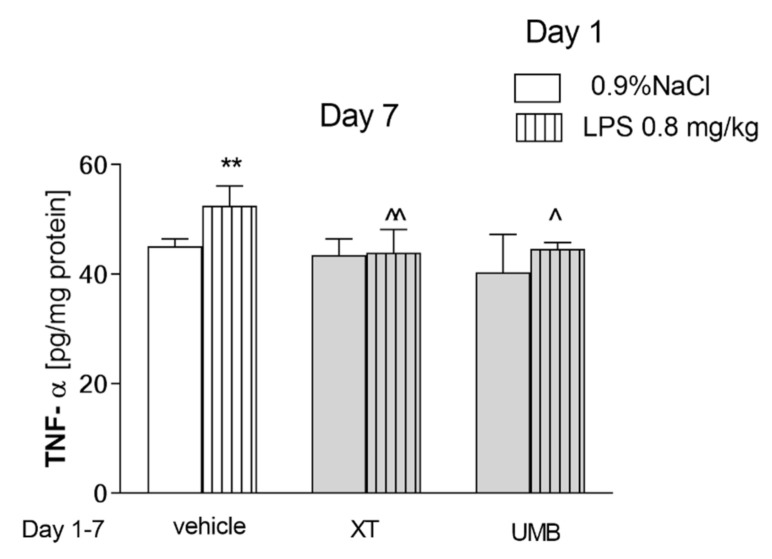
Effects of acute injection of LPS (0.8 mg/kg) and repeated xanthotoxin (XT, 15 mg/kg) and umbelliferone (UMB, 15 mg/kg) alone or in combination on tumor necrosis factor (TNF-α) in the brain. LPS (0.8 mg/kg, i.p.) was administered on the day 1, XT and UMB were administered repeatedly (day 1–7). The brains were collected 24 h after the last injection; *n* = 6; the means ± SEM; ** *p* < 0.01 vs. saline-treated control group, ^ *p* < 0.05, ^^ *p* < 0.01 vs. LPS treated control group; Bonferroni’s test.

Figure 5 presents the effect of acute administration of LPS followed by repeated injection of xanthotoxin and umbelliferone on TNF-α level (pretreatment effect [F(2,36) = 8.29, *p* = 0.0078], and treatment effects [F(1,36) = 7.74, *p* = 0.0021] without interactions [F(2,36) = 2.28, *p* = 0.1202]; two-way ANOVA). Post-hoc Bonferroni’s test showed that LPS administration significantly increased TNF-α level (*p* < 0.01). Both compounds, xanthotoxin and umbelliferone caused a statistically significant decrease in the level of TNF-α in LPS-treated groups (*p* < 0.01, *p* < 0.05, respectively). Both coumarins administered subchronically did not affect TNF-α level in control mice.

Table 1 represents the effect of acute administration of LPS followed by repeated injection of xanthotoxin and umbelliferone on IL-10 level (treatment effect [F(2,36) = 7.27, *p* = 0.0026], without interactions [F(2,36) = 0.46, *p* = 0.6386] and pretreatment effects [F(1,36) = 0.07, *p* = 0.7929]; two-way ANOVA). Although the effect of treatment is considered as significant, post hoc test did not showed any statistically significant effects in IL level.

### 2.4. Effect of Subchronic Injection of Xanthotoxin and Umbelliferone on LPS-Induced Alterations in COX-2

**Figure 6:**
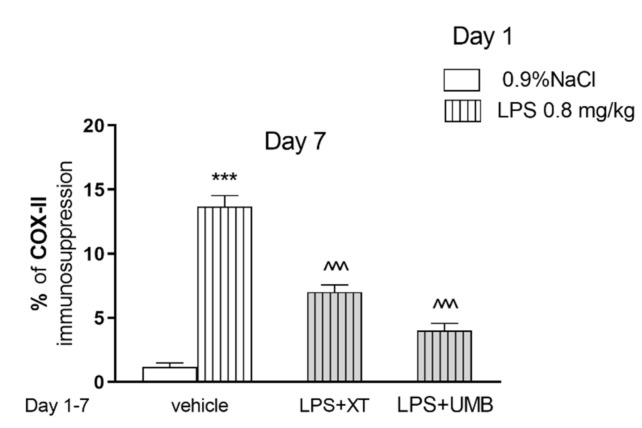
Effects of acute injection of LPS (0.8 mg/kg) and repeated xanthotoxin (XT, 15 mg/kg) and umbelliferone (UMB, 15 mg/kg) in combination on COX-2 immunosuppression in the hippocampus. LPS (0.8 mg/kg, i.p.) was administered on the day 1, XT and UMB were administered repeatedly (day 1–7). The brains were collected 24 h after the last injection; *n* = 3; the means ± SEM; *** *p* < 0.001 vs. saline-treated control group, ^^^ *p* < 0.001 vs. LPS treated control group; Tukey’s test.

**Figure 7:**
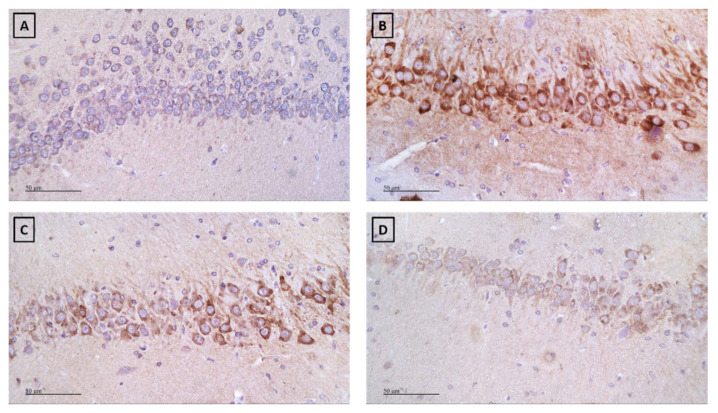
Immunohistochemical staining of COX-2 in the hippocampus (x 400, *n* = 3). (**A**) The saline group showing no expression; (**B**) LPS group showing strong positive expression; (**C**) LPS + Xanthotoxin group showing weak positive expression; (**D**) LPS + Umbelliferone group showing much weaker positive expression (immunopositivity is indicated by brown staining).

Figure 6 and Figure 7 show the influence of coumarins on LPS-induced COX-2 level in mice (one-way ANOVA F(3.11) = 74.13, *p* < 0.001). Post hoc Tukey test showed that LPS increased the percentage of COX-2 expression in the hippocampus in comparison with saline-treated mice, as demonstrated by the strong brown immunohistochemical staining (*p* < 0.001). In contrast, treatment with xanthotoxin or umbelliferone produced significantly weaker positive immunoexpression, reaching approximately 51% (*p* < 0.001) and 29% (*p* < 0.001), respectively, of the LPS group values.

## 3. Discussion

Our studies revealed for the first time the effects of xanthotoxin and umbelliferone on LPS induced memory impairment, as well as inflammatory processes in the CNS in male Swiss mice. Chronic neuroinflammation is a process that underlies many neurodegenerative diseases, e.g., AD, Parkinson’s disease, Inflammatory processes have been implicated in cholinergic or dopaminergic neuron damage in the development and progression of these diseases, respectively [24,25,26]. These complex phenomenon results in cognitive, locomotor and mood impairments observed in different animal models. LPS (endotoxin derived from Gram-negative bacteria) was used to induce cognitive disturbances resulting from a cascade of central neuroinflammation and neurodegeneration in the brain. This neurotoxin induces BBB damage, activates the formation and deposition of Aβ, as well as neuronal loss and microglial activation, and the subsequent release of neurotoxic factors, such as inflammatory cytokines (TNF-α, IL-1β) [27]. Accumulating evidence showed that LPS treatment reduced IL-4 and IL-10 level, while the levels of TNF-α, IL-1β, prostaglandin E2 (PGE2), and nitric oxide (NO) were increased. Also, it was revealed that LPS promoted the expression of cyclooxygenase-2 (COX-2) and inducible nitric oxide synthase (iNOS), as well as activated NF-κB signaling pathway in the mice’s brain [14]. In our pilot study, the doses of 0.5 and 0.75 mg/kg were used for 7 consecutive days. However, we revealed that this subchronic LPS dosage caused high mortality in mice in our laboratory conditions (data not shown). Thus, for presented experiments the acute injection of LPS (0.8 mg/kg, i.p.) was applied, and behavioral and biochemical assays were scheduled on 7 days later.

Our previous studies revealed the procognitive effects of coumarins, imperatorin and xanthotoxin, after both acute and subchronic administration in scopolamine-induced memory deficits model in a well-established PA paradigm [22,23]. It was observed that pre-training administration of coumarins improved acquisition of memory processes.

In this paradigm, scopolamine decreased the index of latency, which is considered as an amnestic effect [22,23]. Scopolamine–induced memory disturbances result from a change in cholinergic neurotransmission, as well as increasing oxidative stress processes [28]. However, LPS-induced amnesia is a more complex process. Thus, the evaluation of coumarins’ effects in this model provides more information regarding the natural products’ procognitive effects. Results obtained in behavioral studies clearly showed that both xanthotoxin and umbelliferone improved memory processes when administered alone. However, only the xanthotoxin improved LPS-induced memory disturbances. Although coadministration of xanthotoxin and LPS decreased the locomotor activity of mice, this effect did not influence evaluated memory processes. The doses of xanthotoxin and umbelliferone were selected based on our previous studies [20]. We chose doses higher than used in studies concerning scopolamine-induced memory impairment [23] because we assumed that the influence of LPS on memory processes would be more complex and strongly pronounced.

Neurodegeneration is usually accompanied by the lower activity of acetylcholine neurons and choline acetyltransferase level in the brain. Also, acetylcholine synthesis, release, and uptake dysfunctions are observed [3]. Therefore, it seemed reasonable to evaluate the level of AChE in the brains isolated from the mice injected with LPS and subsequently treated with coumarins.

In the present study, acute LPS administration significantly increased the AChE level in the mouse brain, but it was alerted by repeated administration of xanthotoxin. In numerous studies, inhibitory properties of coumarins towards AChE were evaluated using Ellman assay. Obtained results were various (IC_50_) depending on the source of cholinesterase origin, starting from 39 µM of xanthotoxin, up to even 720 µM, as an effective dose [18,29]. Umbelliferone showed a similar inhibitory effect on AChE level with IC_50_ equal to 123 µM [30], 145 µM [31]. Furthermore, our previous experiments revealed that xanthotoxin, administered subchronically at the dose of 1 mg/kg, decreased the level of AChE in the scopolamine-induced amnesia model in mice. Although the dose of coumarin was insufficient to influence the level of the enzyme by itself, it effectively decreased this level increased by scopolamine [23]. Present studies confirmed inhibitory effects of xanthotoxin on AChE level, and for the first time showed inhibitory effects of umbelliferone. However, only the xanthotoxin decreases the AChE level elevated by LPS administration. These results are closely correlated with behavioral studies and may suggest the mechanism of the procognitive action of coumarins. Umbelliferone did not improve the memory impairment induced by LPS administration, which may be due to lower inhibition potential against AChE level noticed in in vitro studies.

TNF-α is a potent proinflammatory cytokine with a crucial role in regulating pro- and anti-inflammatory mediators, synthesized by microglia, astrocytes and some populations of neurons as an immune response. TNF-α induced number of intracellular signaling pathways including nuclear factor kappa-B (NF-κB), p38, c-jun N-terminal kinase (JNK), and the ceramide/sphingomyelinase signaling pathway, resulting in a number of responses like releasing other cytokines, prostaglandins and leukotrienes [32,33,34]. This affects the control of processes such as proliferation, cell migration, apoptosis, and necrosis [35]. Uncontrolled reactive oxygen/nitrogen species ROS/RNS production induced by TNF-α decreased antioxidant activity. This process promotes neuronal damage and subsequent inflammation resulting in a feed-forward loop of neurodegeneration [36,37]. Also, in LPS-induced neuroinflammation, TNF-α increases amyloid precursor protein (APP) expression and intracellular Aβ-amyloid level [10,38].

The increase of TNF-α level observed in our study confirmed the role of neuroinflammation in cognitive dysfunction induced by LPS. Accumulating evidence suggests that coumarins exert anti-inflammatory effects in vitro in LPS induced inflammation. The different coumarins extracted from the pomelo peels, a reach source of these compounds, decreased the inflammatory response generated by LPS in RAW 264.7 macrophage cells [39]. Osthole, a simple coumarin with a long aliphatic chain, prevented LPS-induced inflammation in vitro in the mechanisms related to inhibition of NO, PGE2, and IL-6 production in the RAW 264.7 cells [40]. In the same cell model, xanthotoxin exerted anti-inflammatory effects by suppressing iNOS, IL-6, COX-2, and TNF-α expression [41]. Furthermore, bergapten, an analog of xanthotoxin, showed anti-inflammatory effects blocking LPS-induced activation of JAK-STAT, PGE2 production, as well as expressions of iNOS and COX-2 [42]. 

The data mentioned above prompted us to research these compounds’ activity to mitigate the effects of neuroinflammation *in vivo*. A literature search showed that umbelliferone exerts antioxidative effects observed as a decrease of myeloperoxidase (MPO), malondialdehyde (MDA), and increased superoxide dismutase (SOD) activity in lung tissue in the LPS-treated mice. This compound decreases the production of inflammatory cytokines, including monocyte chemotactic protein-1 (MCP-1), IL-6, TNF-α, and IL-1β [43]. Furthermore, another coumarin—osthol relieved the symptoms of ulcerative colitis by reduction of TNF-α level and decrease in the activity of MPO [40]. Regarding CNS activity, it was found that esculetin, a structural analog of umbelliferone, attenuated LPS-induced depressive and anxiety-like behavior in mice and the mechanisms underlying these effects results from the decrease of IL-1β, IL-6, TNF-α level, alleviation of oxidative stress processes and decrease of corticosterone plasma level [44].

In our study, the inflammatory processes observed as an increase of TNF-α level stay in line with the LPS-induced memory impairment observed in the PA test. Both coumarins administered subchronically did not affect TNF-α level in control mice. However, we observed a statistically significant decrease in the level of this factor in coumarins and LPS-treated rodents. These results indicates on anti-inflammatory, TNF-α related mechanisms of procognitive action of xanthotoxin and umbelliferone.

Additionally, IL-10 is a regulatory cytokine with anti-inflammatory properties, which downregulates proinflammatory cytokines, such as IL-1, IL-6, and TNF-α [45]. It is produced by activated immune cells including T cells, B cells, and macrophages. Neuroprotective properties, apart from inhibition of microglial activation, may also result from direct effect on neurons [46]. Increased cellular survival and impact on adult neurogenesis regulation were also reported [47,48]. It seems to be an interesting target implicated in the control of the neurodegenerative process. As a part of counteracting the neuroinflammation, it may become immune-degenerative [49], therefore understanding the molecular mechanisms is crucial for developing new effective therapies.

As the hippocampus, a part of the limbic system, plays important role in cognitive function [50] we decided to evaluate another inflammatory marker—COX-2 in this particular structure. It was revealed that marked COX-2 expression is involved in deleterious events that boost the neurodegeneration cascade and subsequent memory impairment [51]. Moreover, previous studies proved that overexpression of COX-2 is detected in AD patients [52,53]. In the present study, LPS injection caused a profound enhancement in COX-2 protein expression in the hippocampus of mice, which was reversed by xanthotoxin and umbelliferone treatment. These findings suggest the involvement of COX-2 modulation in the anti-inflammatory effect of xanthotoxin and umbelliferone. Furthermore, it was reported previously that xanthotoxin suppresses the COX-2 expression in RAW macrophages [41].

In the present study, we did not observe any changes in the anti-inflammatory factor IL-10 level neither in the LPS-treated group nor coumarins, and LPS administered mice. Thus, the other mechanisms may underlie amelioration of cognitive function observed as a decrease in the index of latency in mice. Although the role of IL-10 in the neuroinflammation is well established, the brain’s dynamic changes may occur within one week after LPS injection. Indeed some authors observed increase of IL-10 after LPS-administration (0.5 mg/kg), but their behavioral tests, as well as sample collection were performed in 1–1.5 h after neurotoxin administration [54,55]. We cannot exclude that IL-10 level elevated immediately after LPS administration returned to the physiological level over the next few days as it is known that the CNS is characterized by high neuroplasticity, and changes occur dynamically.

To sum up, our findings demonstrate that both coumarins improved cognitive functions, but only xanthotoxin significantly enhanced the learning and memory function and reduced the level of AChE in LPS-treated mice. This effect may suggest that only furanocoumarin—xanthotoxin both: attenuates neuroinflammation and enhances cholinergic neurotransmission, thus it can be a potential remedy with procognitive potential effective in treatment of cognitive disorders.

## 4. Materials and Methods

### 4.1. Animals

The experiments were carried out on 8 weeks old naive male Swiss mice (Farm of Laboratory Animals, Warsaw, Poland) weighing 30–35 g at the beginning of the experiments and housed 4 animals per cage. The animals were maintained under standard laboratory conditions (12-h light/dark cycle, room temperature 21+/−1 °C) with free access to tap water and laboratory chow in their home cages, and adapted to the laboratory conditions for at least one week. Each experimental group consisted of 8–10 animals. All behavioral experiments were performed between 8:00 and 15:00 and were conducted according to the National Institute of Health Guidelines for the Care and Use of Laboratory Animals and to the European Community Council Directive for the Care and Use of laboratory animals of 22 September 2010 (2010/63/EU), and approved by the local ethics committee. The different mice were used for each drug and time treatment.

### 4.2. Drugs

The following compounds were tested: LPS (Sigma-Aldrich, St. Louis, MO, USA), xanthotoxin (8-metoxypsoralen), and umbelliferone (7-hydroxycoumarin). Xanthotoxin and umbelliferone were extracted from the dichloromethane extract of the fruits of *Pastinaca sativa* L. and methanol extracts of the fruits of *Heracleum leskovii* Grossch. (Apiaceae), respectively, according to previously published methods [20].

LPS was dissolved in saline solution (0.9% NaCl). Xanthotoxin and umbelliferone were suspended in a 1% solution of Tween 80 (Sigma, St. Louis, MO, USA) and dissolved in saline solution. Drugs were administered intraperitoneally (i.p.) at a volume of 10 mL/kg. New drug solutions were prepared daily. The control group received saline injections of the same volume and via the same route of administration. The doses of umbelliferone, xanthotoxin, and LPS were chosen based on literature data [56], our recently published articles [20,22], as well as preliminary studies.

### 4.3. Behavioral Studies

#### 4.3.1. The Task for the Assessment of Memory-Related Responses

Memory-related responses were measured by the passive avoidance (PA) task [57]. The apparatus and procedure were described in detail in our previous papers [20,23]. The latency time for entering the dark compartment after the last coumarin injection (day 7) was recorded (TL1). Immediately after entering the dark compartment, the food-shock was delivered. In the subsequent trial (retention), the time taken to re-enter the dark compartment was recorded (TL2). No foot-shock was applied in this trial [58,59]. Depending on the used procedure, the PA test allows examining different memory stages according to drug administration time. In our study, drugs were administered before the first trial (before pretest) what interfere with the acquisition of information. 

#### 4.3.2. Spontaneous Locomotor Activity

Opto-Varimex-4 Auto-Track (Columbus Instruments, Columbus, OH, USA) was used to evaluate spontaneous locomotor activity. The device consists of four transparent cages with a lid (43 × 43 × 32 cm), a set of four infrared emitters (each emitter has 16 laser beams), and four detectors monitoring animal movements. Each mouse was placed individually into the cage for 30 min. Animals were randomly allocated into 6 groups to receive injections of saline, xanthotoxin (15 mg/kg), umbelliferone (15 mg/kg), LPS (0.8 mg/kg), or saline/xanthotoxin/umbelliferone co-administered with LPS. 

#### 4.3.3. Treatment

The behavioral experiment was designed to estimate the influence of sub-chronic administration of xanthotoxin and umbelliferone on the acquisition of memory deteriorated by LPS-administration in mice using the PA test. During the sub-chronic administration, xanthotoxin, umbelliferone (15 mg/kg, i.p.), or saline for the control group were administered 60 min after a single injection of LPS (0.8 mg/kg) and then only coumarins were injected once daily for 6 consecutive days. On the seventh day, drugs were administered once (8.00 a.m.), 30 min before the first trial (memory acquisition), and re-tested after 24 h. The locomotor activity was measured immediately after last injection of coumarins for 30 min.

### 4.4. Biochemical Procedures

#### 4.4.1. Tissue Preparation for Cholinesterase and Cytokines Determination

Twenty four h after the last administration of drugs, PA-tested mice were decapitated, brains were collected and placed in ice-cold saline to remove blood. The intact brain without the cerebellum was homogenized in ice-cold PBS (10% *w*/*v*) and centrifuged at 10,000 rpm at +4 °C for 15 min. The supernatant was collected and stored at −80 °C for further analysis. Protein concentration was determined by the Pierce^TM^ BCA Protein Assay Kit. 

#### 4.4.2. Acetylcholinesterase (AChE) Determination

ELISA kit (Cloud-Clone Corp., Houston, TX, USA) was used to AChE determination according to the manufacturer’s protocol. Absorbance was read at 450 nm. The obtained data was presented as ng/mg protein.

#### 4.4.3. Cytokines, TNF-α, and IL-10 Determination

Brain TNF-α and IL-10 were determined by ELISA kits (Biorbyt LLC, San Francisco, CA, USA) and treated according to the manufacturer’s protocol. Absorbance was read at 450 nm. The results presented as pg/mg protein.

#### 4.4.4. Immunohistochemical Detection of COX-2

Isolated hippocampi (*n* = 3) were fixed in 10% (*v*/*v*) formalin for 24 h to perform the immunohistochemistry assay. Paraffin-embedded tissue sections of 3–5 μm thickness were first deparaffinized with xylene and then hydrated in graded ethanol solution and heated in citrate buffer (pH 6.0) for 5 min. Next, the sections were blocked with 5% bovine serum albumin in phosphate buffered saline (PBS) for 2 h. The slides were then incubated overnight at 4 °C with COX-2 rabbit polyclonal antibody (1:100, Thermo Fischer Scientific, Waltham, MA, USA). The slides were rinsed with PBS and incubated for 10 min in a solution of 0.02% diaminobenzidine. Sections were counterstained with haematoxylin, dehydrated, cleared in xylene and then cover slipped for light microscopic examination. Six fields were randomly selected from each section, and positive signals within the section were highlighted, measured, and expressed as the percent area of expression of COX-2 in the immunostained tissue sections using Leica Microsystems (GmbH, Wetzlar, Germany). 

### 4.5. Statistical Analysis

The statistical analysis was performed using one-way or two-way analysis of variance (ANOVA)—for the factors of pretreatment (saline/LPS), treatment (saline/xanthotoxin/umbelliferone), and pretreatment/treatment interactions. 

The data was considered statistically significant at a confidence limit of *p* < 0.05. ANOVA analysis with Tukey’s or Bonferroni’s post-test was performed using GraphPad Prism version 5.00 for Windows, GraphPad Software, San Diego, CA, USA. 

The results obtained in the Spontaneous locomotor activity test were presented as an arithmetic average distance (given in cm) traveled by a mouse ± SEM for each experimental group. For the memory-related behaviors, the changes in PA performance were expressed as the difference between retention and training latencies and was taken as a latency index (LI). LI was calculated for each animal and reports as the ratio:

LI = (TL2 − TL1)/TL1

where TL1—the time taken to enter the dark compartment during the training; TL2—the time taken to re-enter the dark compartment during the retention [60].

## 5. Conclusions

To sum up, in the present study we confirmed that LPS-induced memory impairment is a complex mechanism that mimics the complex pathogenesis of neurodegenerative diseases. Furthermore, we observed for the first time that xanthotoxin, naturally occurring coumarins, induced improvement of cognitive function impaired by LPS injection resulting from a decrease of AChE as well as a decrease of TNF-α level and COX-2 expression. Our study provides support for the treatment of cognitive dysfunction with accompanying neuroinflammation with natural products.

## Figures and Tables

**Table 1:** Effects of acute injection of LPS (0.8 mg/kg) and repeated xanthotoxin (XT, 15 mg/kg) and umbelliferone (UMB, 15 mg/kg,) alone or in combination on interleukin 10 (IL-10) in the brain. LPS (0.8 mg/kg, i.p.) was administered on the day 1st, XT and UMB were administered repeatedly (day 1–7). The brains were collected 24 h after the last injection; *n* = 6; the means ± SEM.

Treatment	Saline	LPS	UMB	XT	LPS + UMB	LPS + XT
IL-10 [pg/mg protein]	38.16 ± 2.217	37.08 ± 1.358	30.11 ± 1.291	37.55 ± 1.408	31.75 ± 2.329	35.76 ± 2.330

## Data Availability

The data presented in this study are available on request from the corresponding author.
